# Highly resolved early Eocene food webs show development of modern trophic structure after the end-Cretaceous extinction

**DOI:** 10.1098/rspb.2013.3280

**Published:** 2014-05-07

**Authors:** Jennifer A. Dunne, Conrad C. Labandeira, Richard J. Williams

**Affiliations:** 1 Santa Fe Institute, 1399 Hyde Park Road, Santa Fe, NM 87501, USA; 2 Pacific Ecoinformatics and Computational Ecology Lab, Berkeley, CA 94703, USA; 3 Department of Paleobiology, National Museum of Natural History, Smithsonian Institution, Washington, DC 20013-7012, USA; 4 Department of Entomology and Behavior, Ecology, Evolution and Systematics Program, University of Maryland, College Park, MD 20742, USA; 5 Microsoft Research, Cambridge CB3 OFB, UK

**Keywords:** food webs, Messel Shale, early Eocene, network structure, niche model, trophic organization

## Abstract

Generalities of food web structure have been identified for extant ecosystems. However, the trophic organization of ancient ecosystems is unresolved, as prior studies of fossil webs have been limited by low-resolution, high-uncertainty data. We compiled highly resolved, well-documented feeding interaction data for 700 taxa from the 48 million-year-old latest early Eocene Messel Shale, which contains a species assemblage that developed after an interval of protracted environmental and biotal change during and following the end-Cretaceous extinction. We compared the network structure of Messel lake and forest food webs to extant webs using analyses that account for scale dependence of structure with diversity and complexity. The Messel lake web, with 94 taxa, displays unambiguous similarities in structure to extant webs. While the Messel forest web, with 630 taxa, displays differences compared to extant webs, they appear to result from high diversity and resolution of insect–plant interactions, rather than substantive differences in structure. The evidence presented here suggests that modern trophic organization developed along with the modern Messel biota during an 18 Myr interval of dramatic post-extinction change. Our study also has methodological implications, as the Messel forest web analysis highlights limitations of current food web data and models.

## 1. Introduction

Comparative analyses of extant food webs have revealed generalities in the underlying network structure of trophic interactions (feeding relationships) among co-occurring taxa regardless of habitat [1–5]. The question of whether such generality extends to ancient ecosystems remains open, as previous food web studies of Late Cretaceous terrestrial assemblages [6], Permian and Triassic terrestrial assemblages [7] and Cambrian marine assemblages [8] used low-resolution, high-uncertainty data that were highly space and/or time-averaged. The Cambrian study reported similarities in ancient and extant structure based on webs with 50 or fewer taxa. The Late Cretaceous and Permian–Triassic studies assumed similar structure to extant webs as a way to create instances of possible palaeofoodwebs from aggregated, guild-level fossil data [6,7]. Here, we present and analyse new food web data comprised of thousands of highly resolved, spatially and temporally constrained feeding interactions among hundreds of aquatic and terrestrial taxa representing probably the best preserved post Cretaceous–Palaeogene (K–Pg) continental biota, the 48 Myr-old Messel deposit.

Profound environmental events and their biotal effects occurred in the 18 Myr between the end-Cretaceous mass extinction and the deposition of the Messel assemblage. Evidence for ecologically unbalanced food webs several million years into the Palaeocene aftermath of the K–Pg boundary crisis [9] comes from exceptionally depauperate floras [10,11], plant–insect interaction data reflecting severely unbalanced ecosystems [12], unstable mammalian community structure [13], and reptile and bird gigantism amid elevated greenhouse conditions that approached lethality in some tropical regions [14,15]. A second, early Eocene series of events was characterized by a sudden spike in significantly elevated temperatures and *p*CO_2_ levels of the Palaeocene–Eocene Thermal Maximum (PETM) [16,17] that induced new types and high levels of insect herbivory [18]. Soon after this 10^5^ year-long spike, major biogeographic and taxic transformation of the biota ensued, such as major latitudinal shifts of floras [19], penecontemporaneous diversification of termite [20], ant [21] and bee [22] social insect clades, and replacement of Palaeocene-type warm-blooded vertebrates by new major lineages of Eocene birds [23] and mammals [13,24] that persist to the present day.

These diversification events continued during a prolonged, multi-million-year-long intensification of greenhouse conditions at the Early Eocene Climatic Optimum (EECO), in which temporally constrained pulses of elevated temperature and *p*CO_2_ levels and various Eocene Thermal Maxima rivalled that of the PETM [16]. During the EECO and immediately prior to Messel deposition, another biotal transformation occurred concomitant with a greenhouse-to-icehouse shift in the physical environment, signalled by climate cooling and freshwater flooding of the Arctic Ocean. This shift was attributable to major effects of oceanic heat transport reflected in the Azolla Event [25]. These K–Pg to Messel events (electronic supplementary material, table S5) likely affected food web structure, particularly for Northern Hemisphere ecosystems, that would represent a major, cumulative effect of the previous 18 Myr and not the subsequent 48 Myr of ecosystem change reflected in modern ecosystem structure. As recently summarized in a prominent review, which referenced the K–Pg crisis, ‘Mass extinction events may continue to affect the structure of biotic interactions long after ecosystems have recovered to pre-extinction diversity levels’ [26, p. 499]. These factors suggest that it is an open question whether Messel food web organization would be similar or anomalous in comparison to extant web structure.

The Messel lacustrine deposit, near Darmstadt, in central Germany, is of latest early Eocene age, based on radioisotopic dates [27]. The maar lake strata is part of the Messel Formation and consists of 190 m of oil shale in a 0.7 km^2^ basin [28] that represents from 1.0 to 1.6 Myr of time [29], with 0.6 Myr of this time providing a sedimentologically and climatically circumscribed record [30] (electronic supplementary material, methods S1). The Messel biota records a considerably abbreviated geologic window of deposition both temporally and spatially (electronic supplementary material, methods S2), increasing the likelihood of ecological co-occurrence and interactions of preserved species. Compared to 14 other well-documented and exceptionally preserved fossil deposits [31], Messel's spatio-temporal confinement lies within the top two or three, exceeded by narrower spatial extent, temporal resolution or both, only by the Cambrian Burgess Shale, Devonian Rhynie Chert and Pleistocene Rancho La Brea asphaltum deposits.

Detailed species and feeding interaction data compiled for the Messel system were used to create two habitat-specific lake and forest food webs to ensure spatial co-occurrence and potential interaction among taxa within each food web. The Messel webs were compared to food webs from extant habitats, in particular food webs that portray cumulative interactions over time, using model-based analyses of several aspects of network structure. Simple models like the niche model generate food web structure similar to that observed for empirical webs [32,33]. Here, following the approach of a previous study [34], we use the niche and other models as normalization tools whose output provides ways to compare the structure of food webs with variable numbers of species and links, given the well-documented scale dependence of food web structure on diversity and complexity of the web [2–4,34–37]. The fits of the models are also scale-dependent, generally showing significantly decreasing fit with increasing species richness or simple measures of complexity [33,34,38–40]. This provides an additional way to compare network structure of food webs with very different numbers of species and links, as is the case in this and a previous study [34].

## 2. Material and methods

### (a) Species and trophic links

The Messel dataset [41] includes all documented species deposited within the small maar lake basin, including taxa from the lake's water column and benthos and from the immediately surrounding paratropical forest [27,29,30]. The Messel deposit provides a remarkably comprehensive record of taxa from all trophic levels (electronic supplementary material, methods S3). Evidence used to assign trophic links between Messel taxa came from 10 lines of indirect to direct observations: (i) taxonomic uniformitarianism, (ii) functional morphology, (iii) gut contents, (iv) damage patterns, (v) stratigraphic co-occurrence, (vi) body size, (vii) coprolites, (viii) host relationship, (xi) chemical and isotopic signatures and (x) ichnological evidence (electronic supplementary material, figures S1–S5) [41]. For each evidence category, one or more operational guidelines from ecology or palaeoecology were used to infer a link between a consumer and a resource taxon. These 10 categories have been used informally throughout the modern and fossil continental record for inferring trophic roles of organisms [42–46]. More detailed description of the lines of evidence with particular reference to the Messel ecosystem can be found in the electronic supplementary material, methods S4.

The 10 lines of evidence were used to assign one of three certainty levels to each inferred link [41]. We assigned the highest certainty level when three or more categories provided evidence for a link, or when gut contents were identified. The intermediate certainty level was assigned when two categories applied and the lowest certainty level corresponded to only one category of evidence. By operationalizing the assignment of links with explicit guidelines, assumptions used to establish the certainty of links were kept to a minimum.

### (b) Food webs

A lake web was extracted from the full Messel web by eliminating links that involve solely terrestrial organisms. The remaining data were purged of dangling animals that lack food chains connecting them to at least one aquatic basal taxon, as well as any resource or consumer links of those animals. A forest web was similarly generated, but instead eliminated links that involve entirely aquatic organisms. Taxa that occur in both habitats at some point in their life cycle can appear in either or both habitat-specific webs, as long as they retain at least one food chain connecting them to a basal taxon in that habitat. Reduced, higher certainty versions of all three Messel webs were constructed by eliminating low-certainty links and purging the datasets of dangling animals and their links. We generated ‘trophic species’ versions of each web by aggregating taxa with the exact same set of predators and prey [47]. Following convention [1–5,8,32–35], we focused analyses on trophic species webs for the Messel lake and forest food webs and 30 extant habitat-specific webs used in previous comparative structural studies (electronic supplementary material, table S1). We excluded extant food webs based on narrowly constrained temporal sampling (e.g. webs based on gut contents sampled on one or a few days), focusing on webs that represent cumulative, likely trophic interactions among taxa that co-occur and have the possibility of interacting, similar to the nature of the Messel lake and forest food webs.

### (c) Analyses

We analysed several commonly studied aspects of food web structure to provide a broad comparison of the trophic organization of Messel and extant webs. We used a set of three modelling frameworks and associated analyses described in more detail elsewhere [34]. First, we tested the fit of cumulative resource (generality) and consumer (vulnerability) distributions of each food web to predictions of a null model based on maximum information entropy (MaxEnt) [38]. We tested the fit of MaxEnt predictions (*n* = 1000) to empirical distributions by calculating goodness of fit, ƒ_G_, and relative width of the degree distribution, *W*_95_, specifically the width of the resource distribution *Res W*_95_ and of the consumer distribution *Con W*_95_. When ƒ_G_ ≤ 0.95, the empirical web's degree distribution does not differ significantly from the model distribution at the 95% confidence interval. When −1 ≤ *W*_95_ ≤ 1, the empirical distribution is neither significantly narrower (*W*_95_ < −1) nor broader (*W*_95_ > 1) than the distribution predicted by the model at the 95% confidence interval.

Second, we calculated 14 network structure properties [32] whose definitions and significance are discussed elsewhere (table 1, Metrics 6–19 in [34]). The properties are the fractions of top, intermediate and basal species (*Top*, *Int* and *Bas*); the fractions of cannibals, herbivores, omnivores and species in loops (*Can*, *Herb*, *Omn* and *Loop*); the standard deviations of normalized total links, generality and vulnerability (*LinkSD*, *GenSD* and *VulSD*); the mean short-weighted trophic level (*SWTL*); the mean maximum species trophic similarity (*MaxSim*); the mean shortest path length (*Path*) and the mean clustering coefficient (*Clus*). We generated 1000 niche model webs [32] with the same *S* and *C* as the empirical web, and for each property calculated model error (ME), the normalized difference between the model's median value and the empirical value [33]. ME > |1| indicates that the empirical property falls outside the most likely 95% of model values, with negative and positive MEs indicating model underestimation and overestimation of the empirical value, respectively. While these properties are not independent [37], we are primarily concerned with systematic patterns of change in average ME, as in prior studies [8,33,34].

**Table 1.** Basic properties of the Messel Shale food webs. (*S*, number of taxa; *L*, number of trophic links; *L*/*S*, linkage density and *C* (*L*/*S*^2^), directed connectance. Cert-Low, Cert-Int, Cert-High indicates the percentages of links that are low, intermediate or high certainty. ‘Red.’ refers to reduced web versions that exclude low-certainty links and associated taxa. ‘Tro.’ refers to trophic species web versions.)

web version	*S*	*L*	*L/S*	*C*	Cert-Low	Cert-Int	Cert-High
full	700	6444	9.21	0.013	22.4	31.9	45.7
forest	630	5534	8.78	0.014	22.3	32.0	45.7
lake	94	517	5.50	0.059	27.6	30.8	41.6
full Red.	630	4602	7.30	0.012	0	39.0	61.0
forest Red.	557	3885	6.97	0.013	0	38.5	61.5
lake Red.	90	370	4.11	0.046	0	43.0	57.0
full Tro.	700	6444	9.21	0.013	22.4	31.9	45.7
forest Tro.	629	5530	8.79	0.014	22.3	32.0	45.7
lake Tro.	93	508	5.46	0.059	28.0	30.5	41.5
full Red. Tro.	630	4602	7.30	0.012	0	39.0	61.0
forest Red. Tro.	556	3881	6.98	0.013	0	38.5	61.5
lake Red. Tro.	88	360	4.09	0.046	0	43.3	56.7

Third, we used a probabilistic niche model (PNM) [39] to parametrize the niche model directly against each food web. The PNM produces a maximum-likelihood estimate (MLE) of the fundamental niche model parameters for each species *i* in a given web: its niche position *n_i_*, its optimal feeding position *c_i_* and its feeding range *r_i_*. This allows computation of the probability of each link in a web according to the model, and the overall expected fraction of links in a web predicted correctly (ƒ_L_).

Model results for extant webs were taken from prior studies, resulting in a set of 30 extant webs for MaxEnt and PNM analyses [38,39] and a subset of 11 extant webs for niche model analyses [33] (electronic supplementary material, table S1). We also used the previously documented systematic decreases in model fit with food web species richness or links per species [33,34,38–40] as a further means of comparing webs of varying diversity and complexity, an especially crucial issue for the highly diverse Messel forest web.

## 3. Results

**Figure 1.**
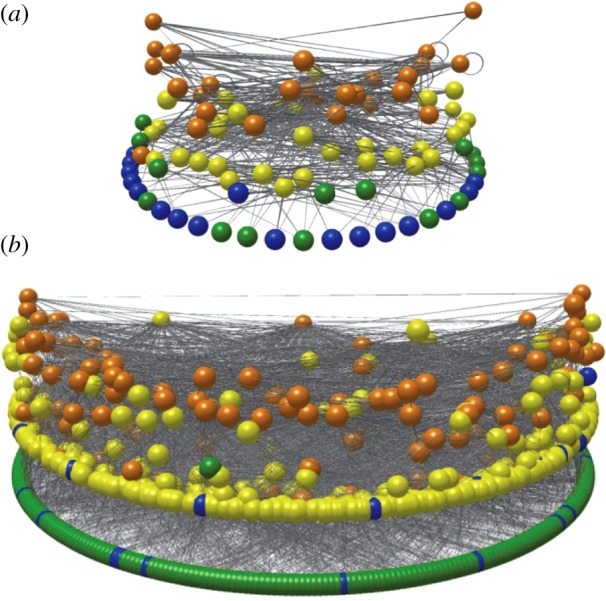
Visualizations of the Messel lake and forest food webs. (*a*) Lake food web and (*b*) forest food web. Spheres represent taxa, lines represent feeding links. Links that loop indicate cannibalism. The vertical axis corresponds to short-weighted trophic level [48], with autotrophic taxa and detritus at the bottom level. Images produced with Network3D [49,50]. Colours of nodes correspond to taxonomic affiliation of species. Green, plants, including algae and diatoms; blue, bacteria, fungi and detritus; yellow, invertebrates; orange, vertebrates.

The full Messel food web consists of 700 trophically unique taxa and 6444 links (electronic supplementary material, figure S6) [41]. Fifty-four per cent of the taxa are resolved to the genus or species level, and 82% to the family level or better. The taxa consist of 187 plants (principally vascular plants), 326 invertebrates (overwhelmingly insects), 143 vertebrates and 44 protists, fungi and prokaryotes. Seventy-eight per cent of the links have intermediate or high certainty (table 1). The full Messel web was split into a Messel lake web with 94 taxa and 517 links and a Messel forest web with 630 taxa and 5534 links, with 78% and 72% intermediate plus high-certainty links, respectively (table 1 and figure 1). Only one pair of taxa in each habitat web shares the same set of predators and prey, resulting in trophic species versions of the webs with 93 (lake) and 629 (forest) taxa. Reduced, higher certainty versions of the Messel food webs that exclude low-certainty links have 4–12% fewer taxa and 28–30% fewer links (table 1). The two habitat-specific webs have similar maximum generality, as each contains a taxon that feeds on *ca* 30% of taxa. However, the Messel forest web has a greater incidence of trophic specialization, with 14% of taxa feeding on one taxon compared with 5% specialists in the Messel lake web.

Almost all aspects of the trophic structure of the Messel lake web fit within the ranges observed for extant webs, including its trophic species richness *S* of 93 and connectance *C* (the proportion of possible links that are realized, *L/S^2^*) of 0.059 (table 1; electronic supplementary material, table S1). The consumer distribution of the Messel lake web is marginally significantly narrower than a null MaxEnt expectation (electronic supplementary material, figure S7 and table S2), but five of 30 extant webs have narrower distributions. The resource distribution width is not significantly different than the MaxEnt expectation, as is the case for 24 of 30 extant webs. The mean absolute niche ME for 14 metrics of the Messel lake food web is exceeded by three of 11 extant webs (electronic supplementary material, table S2), and the number of individual metrics poorly fit by the niche model in the Messel Lake web (six of 14) is equalled or exceeded by three extant webs with 6, 8 and 12 poorly fit metrics (electronic supplementary material, table S3). Considering particular metrics, only the proportion of taxa that are basal (Bas) displays an ME for the Messel lake web that falls outside the range of ME across the extant webs (−1.80, indicating model underestimation), although two extant webs have similar ME of −1.50 (electronic supplementary material, table S3). The PNM correctly predicts 52.4% of Messel lake links, which is near the low end but within the range for 30 extant webs (electronic supplementary material, table S2).

**Figure 2.**
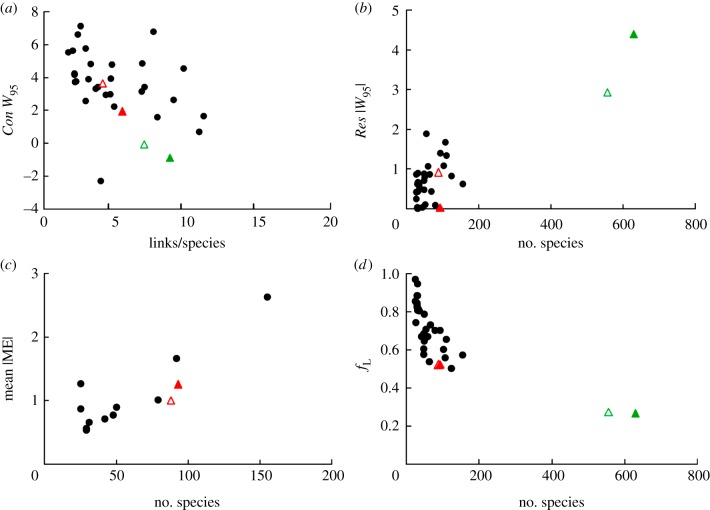
Scale dependence of MaxEnt, niche model and PNM results. (*a*) Relative width of the empirical consumer distributions (*Con W*_95_) plotted against link density (*L/S*). (*b*) Absolute value of the relative width of the empirical resource distributions (*Res* |*W*_95_|) plotted against species richness (*S*). (*c*) Mean absolute niche model error (|ME|) plotted against *S*. (*d*) The fraction of links correctly predicted by the PNM (*f*_L_) plotted against *S*. Black circles show results for extant food webs. Green and red triangles show results for the Messel forest and lake food webs, respectively, with open triangles indicating results for reduced web versions.

An assessment of where Messel lake web structure falls compared to extant webs given linear scale-dependent trends of model fit strengthen this assessment of similarities in network structure. For extant webs, the absolute width of the resource distribution (*Res* |*W*_95_|) (figure 2*b*) and the absolute mean niche model error (|ME|) (figure 2*c*) significantly increase with *S* (electronic supplementary material, table S4). The fraction of links correctly predicted by the PNM (*f*_L_) (figure 2*d*) significantly decreases with *S*, and the width of the consumer distribution (*Con W*_95_) (figure 2*a*) significantly narrows with increasing *L/S* (electronic supplementary material, table S4). The Messel lake web, with relatively high *S* and intermediate *L/S*, fits within those trends (figure 2), and its addition alters linear regressions based on extant webs minimally (electronic supplementary material, table S4).

Comparing the Messel forest food web to extant webs is more challenging than for the Messel lake web. With *S* of 629 and *C* of 0.014, the Messel forest web has far greater trophic species richness than the largest extant web (*S* = 155) and lower connectance than the lowest extant web (*C* = 0.034) (table 1; electronic supplementary material, table S1). Its high *S* and low *C* are likely attributable to very high resolution of 185 terrestrial plants, 323 mostly insect herbivores and their trophic interactions, many of which are specialized. All extant terrestrial web datasets and most aquatic datasets have low resolution of plant taxa, invertebrate taxa and/or their interactions. For example, a Caribbean reef web with 249 taxa, not included in this analysis owing to highly uneven resolution [3], has fish identified mostly to species comprising 84% of taxa, and invertebrates and primary producers highly aggregated in groups comprising 14% and 2% of taxa, respectively [3,51]. However, it is also possible that lower connectance in the Messel forest web is in part an artefact of incomplete detection of all fossil plant–insect trophic interactions.

Analysis of the structure of the Messel forest web reveals a serious limitation of the niche model [32], which cannot generate webs that are single connected networks for webs with high *S* and low *C*. This limits possible Messel forest web comparisons to link distributions and link probabilities. The Messel forest consumer distribution is significantly narrower than the MaxEnt expectation, and narrower than all but one extant web (electronic supplementary material, table S2 and figure S7). However, this level of narrowness fits well with the significant trend of decreasing width of *Con W*_95_ with increasing *L/S* (figure 2*a*; electronic supplementary material, table S4), given the Messel forest *L/S* of 8.79. The absolute width of the resource distribution (*Res* |*W*_95_|) of the Messel forest web is significantly wider than both the MaxEnt expectation (electronic supplementary material, figure S7) and extant web distributions (figure 2*b*), and the PNM only predicts 26.6% of Messel forest links correctly (*f*_L_), compared with the lowest value of 50.2% for an extant web (figure 2*d*; electronic supplementary material, table S2). As both the *Res* |*W*_95_| and *f*_L_ display significantly decreased model fit with increasing *S* (figure 2*b,d*; electronic supplementary material, table S4), and the Messel forest has high *S*, it is unsurprising that there are strong differences compared with results for webs with *S* < 160. Indeed, the position of the Messel forest's *Res* |*W*_95_| and *f*_L_ in relation to *S* look like simple extensions of linear relationships observed for webs with *S* < 160 (figure 2*b,d*), although significance tests are not justified given the large gap between the Messel forest data point and the other data points. Results for the higher certainty, reduced versions of the Messel lake and forest webs are consistent with observed trends in structure across Messel and extant webs (figure 2; electronic supplementary material, table S2), similar to earlier studies which found that network structure patterns are robust to removals of approximately 20–30% of links and associated taxa [8,52]. It also is likely the case that both Messel and extant food web data fail to include some links and species that actually occurred. However, food web structure has been shown to be robust to the addition of approximately 20% of species and links [52].

To better assess the impact of plant–insect interaction resolution on Messel forest food web resource distribution width (*Res W*_95_) and the fraction of links correctly predicted by the PNM (*f*_L_), we turned to highly resolved and well-sampled species interaction data from Norwood Farm, UK [53]. While not representing a full food web, a subset of the dataset includes interactions between 95 plants and 312 herbivores which are potentially comparable to interactions between the 185 plants and 323 herbivores in the Messel forest web. In both cases, the consumers are mostly insects. The trophic species version of the Norwood bipartite herbivore–plant subweb has *S*_plant_ = 91, *S*_consumer_ = 146, *L* = 939 and *C* = 0.07, where *C* is calculated as *L*/(*S*_plant_ × *S*_consumer_), while the Messel subweb has *S*_plant_ = 185, *S*_consumer_ = 294, *L* = 2127 and *C* = 0.04. The connectances of these two subwebs do not differ greatly, suggesting that the low connectance of the full Messel forest food web may reflect the attribute of high incidence of specialization between insect herbivores and plants more than the artefact of low sampling of trophic interactions among a highly resolved set of plants and insects.

Analysis of the link distributions indicates that both subwebs have similar, and significantly broader resource distributions than the MaxEnt expectation (Messel *Res W*_95_ = 2.93, Norwood *Res W*_95_ = 3.11). This suggests that the apparent outlier status of *Res W*_95_ of the full Messel Forest web is likely attributable to its high resolution of plant–insect interactions. However, the PNM correctly predicts 52% of Norwood links but only 26% of Messel links. The Norwood data include about half as many taxa and links as the Messel data, which suggests that much of the difference in *f*_L_ may be attributable to strong scale dependence of PNM fit with *S*, observed for full food webs [39] as well as for smaller herbivore–plant subsets of extant webs [40]. In addition, the Norwood data, with a focus on aphids, nectar/pollen feeders and seed consumers, lacks trophic groups well-represented in the Messel data such as folivores which may affect *f*_L_. Future compilation of comprehensive, highly resolved food web data for modern systems, particularly terrestrial systems with diverse insect–plant interactions, will help fill in current data gaps and allow more definitive comparisons of Messel forest and extant web structure.

## 4. Discussion

The Messel biota represents ecologically modern lake and forest taxa occurring after a series of major physical disruptions and biotal reorganizations 18 Myr after major ecosystem change at the K–Pg boundary [9–15] and subsequent significant modification that continued throughout the Early Eocene [16–26] (electronic supplementary material, table S5). The evidence presented here suggests that the structure of feeding interactions among Messel taxa was also modern despite the major changes of the preceding 18 Myr and was also apparently robust to the subsequent 48 Myr of species turnover and evolution.

There is unambiguous evidence for the similarity of Messel lake food web structure to that of extant webs. Comparisons of resource and consumer distributions, food web metrics and the fraction of links correctly predicted by the PNM place Messel lake web structure well within ranges observed for extant webs. Those findings are strengthened by evidence based on the scale dependence of model fit with species richness *S* and link density *L/S*, which shows that Messel lake food web results are consistent with significant linear trends in model fit observed across extant webs.

The evidence for the similarity of the Messel forest web is suggestive but necessarily less definitive due to its much greater species diversity and lower connectance than extant webs and the Messel lake web, which allows for less direct comparisons of detailed structure. Deviation of link distributions from a MaxEnt expectations and the fraction of links correctly predicted by the PNM indicate that Messel forest web structure falls outside what is observed for extant webs and the Messel lake web. However, the scale dependence of the fit of those models indicate either that Messel forest structure fits well within observed scale-dependent trends in the case of consumer distribution width (as a function of *L/S*) or appears to be a result of decreased fit with increasing species richness (i.e. absolute width of the resource distribution, fraction of links predicted by the PNM). These results, as well as a comparison of the herbivore–plant subnetworks of the Messel forest and Norwood farm webs, suggest that apparent differences in Messel forest web structure are likely attributable primarily to its very high resolution and diversity compared to other webs. This heightened characterization of the Messel forest web results from a detailed accounting of plant–invertebrate interactions thus far missing from extant food webs. Future work to further elucidate similarities and differences in Messel forest and extant terrestrial web trophic organization could include comparisons of other kinds of plant–insect subwebs, for example, those that focus on herbivores and their parasitoids and/or predators for a suite of plants or for a particular plant [54–56].

Extant webs display fundamental similarities in trophic organization across habitats [3,4], despite different kinds of organisms filling similar ecological niches (e.g. large predatory fishes versus mammalian carnivores). There are also ecological differences in some trophic habits and niche-filling in the Messel forest versus extant ecosystems. For example, Messel *Eomanis* anteaters and *Propalaeotherium* horses have very different diets than their modern descendants, and large ground-dwelling carnivorous birds occupied ecological niches filled today by top mammalian predators. But as with extant webs, such substitutions are likely not associated with fundamental changes in overall trophic organization.

Our analysis of the Messel forest web highlights limitations in empirical and modelling aspects of current food web structure analysis. The webs previously used to evaluate network structure have species richness less than 160, and in most cases have uneven resolution with highly aggregated invertebrates and basal taxa and highly resolved vertebrates. The Messel forest food web introduces new empirical benchmarks for high diversity, high resolution of terrestrial plant–insect interactions, and documentation of adjoining, interlinked habitats. Other highly and evenly resolved food webs with *S* of 500–700 are needed for more direct comparisons of Messel forest and extant trophic organization and to corroborate or reject our hypothesis of similar structure. In addition, new data with *S* > 100 are needed to fill in gaps in our understanding of the scale dependence of food web structure and model fit. Regarding the simple models used here, they appear to fit the structure of food webs with *S* < 100 reasonably well, but that fit decays rapidly, systematically and linearly with increased *S*. Also, models like the niche model [32] cannot be tested against very high diversity, low connectance webs like the Messel forest owing to difficulties in generating single connected component networks for comparison. Our analyses of the Messel forest web suggest that more diverse and highly resolved data require development and testing of new network structure models, and may require a shift from low- to higher dimensional approaches.

The compilation of trophic information for other fossil assemblages can provide opportunities to explore other aspects of the stability of ecosystem organization over various geologic timescales [6–8]. For example, it has been suggested that food web analysis can be used to look for changes in trophic organization [8] and robustness [6,7,57] before, during and/or after major extinction events. We have listed nine target deposits comparable to Messel that span the 18 Myr interval prior to Messel, and extend downward to the Late Cretaceous (electronic supplementary material, table S5). Although food webs of these freshwater and terrestrial ecosystems remain unknown immediately preceding the K–Pg ecological crisis and the Palaeogene prior to Messel, several major environmental disruptions did occur, notably the PETM [16], several EECO pulses [17] and the Azolla Event [25] (electronic supplementary material, table S5). These major pulses involving mostly palaeoclimatic change evidently had major consequences in altering food web structure prior to Messel. A food web analysis of a latest Cretaceous lake deposit ecologically analogous to Messel would reveal whether food webs after the K–Pg crisis lacked significant trophic reorganization [58,59], or underwent rapid and possibly permanent change associated with a prolonged recovery [11–13]. The latter would have entailed a several million-year interval of significant habitat and community reorganization, fostering regional trophic cascades that may have resulted in changes in food web properties such as connectance, trophic level, path length and link distributions, which reflect the balance of specialization and generality of feeding habits, and the balance of vulnerability and invulnerability to predation.

Successive analyses of trophic structure over deep time (electronic supplementary material, table S5) could potentially be used to assess other interesting questions. We provide two examples. First, are there similarities between ecological assemblies of food webs at very short timescales following localized perturbations versus evolutionary assembly of food webs at very long timescales following major extinction crises? A recent study of the dynamics of food web structure on defaunated mangrove islets showed an increase in proportion of species that are specialists and a decrease in connectance over 2 years' reassembly time [60]. It may be the case that evolutionary assembly of food webs over geologic time following an extinction crisis would also tend to favour generalists at early stages and specialists at later stages, altering link distributions and connectance as well as other properties. In addition, researchers have begun to explore models of food web development based on simple evolutionary and trophic dynamics, but have not had data to compare to their developing structure [61].

A second issue is the question of whether there is evidence for changes in trophic structure that indicate evolutionary escalation [62] such as a Red Queen driver [63]. Escalation typically is portrayed as an evolutionary pattern captured by measurement of phenotypic response to predation pressure, such as shell thicknesses and drill-hole frequencies evaluated in stratigraphic time series [64]. Historically, escalation studies have minimally referenced the ecological underpinnings that determine patterns of phenotypic response of prey to predators. Identification and temporal tracking of particular predator species and their compartmentalized predator–prey subwebs within a larger food web could provide evidence for ecological adjustments resulting in escalation phenomena predicted by Red Queen evolutionary theory [65], or could support the counter argument that given the normal context of multi-species interactions, the Red Queen hypothesis does not hold in most cases [66,67].

In summary, there are many possible opportunities to compile, analyse and model detailed ecological network data for ancient ecosystems. Such data can potentially be used to address a wide array of questions such as the generality of food web structure through deep time [8], the stability and robustness of ecosystems to major perturbations [6,7], macroevolutionary dynamics from an ecological interaction perspective [67] and the assembly of ecosystems at multiple spatial and temporal scales. This study, while not the first to consider ancient food webs, does give the first example of highly resolved, high-certainty, comprehensive ecological network data for ancient ecosystems. It also provides the beginning of an empirical foundation for addressing a variety of important questions at the interface of palaeobiology, ecology and evolution.

## Data accessibility

Data underlying this research are publicly available at Dryad doi:10.5061/dryad.ps0f0 [41].
